# Osteoporotic Vertebral Fractures: An Analysis of Readability and Quality of Web-Based information

**DOI:** 10.7759/cureus.26029

**Published:** 2022-06-17

**Authors:** Yasir Hidayat, Ashley Ghanshyam Rajkoomar, Muhammad Abrar Qadeer, Lester G D’Souza

**Affiliations:** 1 Orthopaedics and Trauma, University Hospital Limerick, Limerick, IRL

**Keywords:** orthopedic surgery, online health information, osteoporotic spinal fractures, fragility spinal fractures, osteoporotic vertebral fractures

## Abstract

Introduction

Vertebral compression fractures are among the most common fragility fractures with significant morbidity and mortality. With an aging population, the incidence of these fractures is on the rise. In this age of social and electronic media, there is a plethora of online information available. While access to healthcare information has increased, most of these websites remain beyond the comprehension of their target audience.

Objective

To assess the readability and quality of online information regarding osteoporotic vertebral fractures.

Methods

A search for the terms osteoporotic vertebral fractures, osteoporotic spinal fractures, and fragility spinal fractures was performed using the top five search engines. Eighty-three websites were identified and analyzed. Quality assessment was done using the DISCERN and Journal of the American Medical Association (JAMA) tools while readability was analyzed using the Flesch Reading Ease Score (FRES), Flesch Kincaid Grade (FKG), and Gunning Fog Index (GFI).

Results

The mean DISCERN score was 39.55 while the mean JAMA was 2.2. Readability testing revealed a mean FRES score of 49.26 with 16 websites having a score of > 60, FKG 8.38, and GFI of 9.51. 33 websites had an FKG score of 8 or below 8.

Conclusion

The above results indicate that web-based information relating to osteoporotic vertebral fractures is of variable quality and readability. Although 40 % of websites are at the eighth grade or below level, only 16 % of websites are above the FRES score of 60, which makes online information difficult to comprehend by an average patient.

## Introduction

With 91% of households having access to the internet in Ireland, patients frequently consult the internet for health information. In 2020, 60% of people used the internet to seek information related to health and in comparison to preceding years, there has been an increase of 3% [1]. Health-related information has also significantly increased over the past years [2]. As of 2009, 72% of users in Ireland with a high degree of education looked for health advice online [3].

Health literacy is a significant predictor of health status and outcome. Low health literacy has been associated with poor compliance behavior and increased hospitalization rates [4]. Health literacy is the degree to which individuals can search, process, and use the information to inform health-related decisions and actions for themselves and others [5].

Osteoporosis is one of the most common metabolic bone disorders worldwide [6]. It is a silent disorder that does not manifest itself until a fracture occurs. Osteoporosis causes more than 8.9 million fractures annually worldwide. This number is on the constant rise, as this relates in part to the increased longevity of the population [7].

Vertebral compression fractures occur secondary to minimal or moderate axial trauma [8]. Their incidence is not well-documented because of the non-specific nature of the symptoms mimicking other causes of back pain. It has been reported that an estimated 1.5 million vertebral compression fractures occur every year in the U.S. [9].

Vertebral fractures may give rise to pain, loss of height, and progressive kyphosis, which will result in further deterioration in activities of daily living. Severe deformities, give rise to cardiac, respiratory, and gastrointestinal disorders [10]. The morbidity from an acute vertebral compression fracture is comparable to a hip fracture and is associated with an increase in mortality rate [11].

While there is an abundance of information available online on osteoporosis, there appears to be a significant lack of easily readable and quality information on vertebral compression fractures. This is even though the recommendation is that educational material should not be above the literacy level of a sixth-grade level student [12]. Keeping these facts in mind, we decided to evaluate the available information. The purpose of our study is to review online resources that are accessible to patients and to perform an objective assessment of internet-based education materials related to vertebral compression fractures.

## Materials and methods

**Figure 1:**
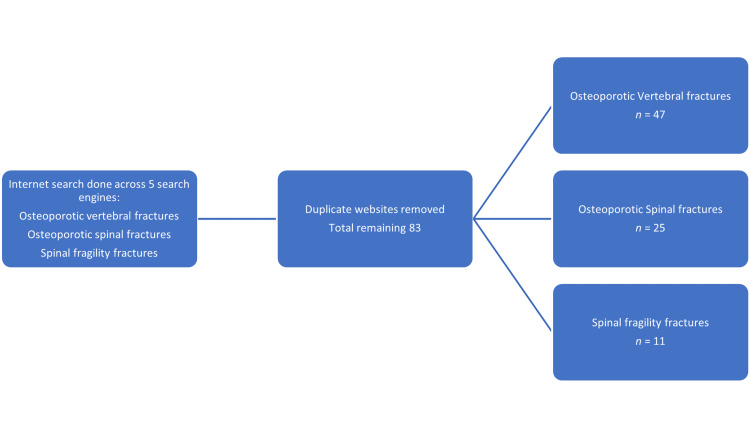
Flowchart demonstrating internet search methodology

A search of three keywords, osteoporotic vertebral fractures, fragility spinal fractures, and osteoporotic spinal fractures, was performed using the top five internet search engines on the 29th of January, 2022. These search engines account for more than 99% of the market share as of April according to www.statcounter.com [13]. The first five pages from each search engine were taken and 83 websites were identified after removing the duplicate sites (Figure 1).

**Figure 2:**
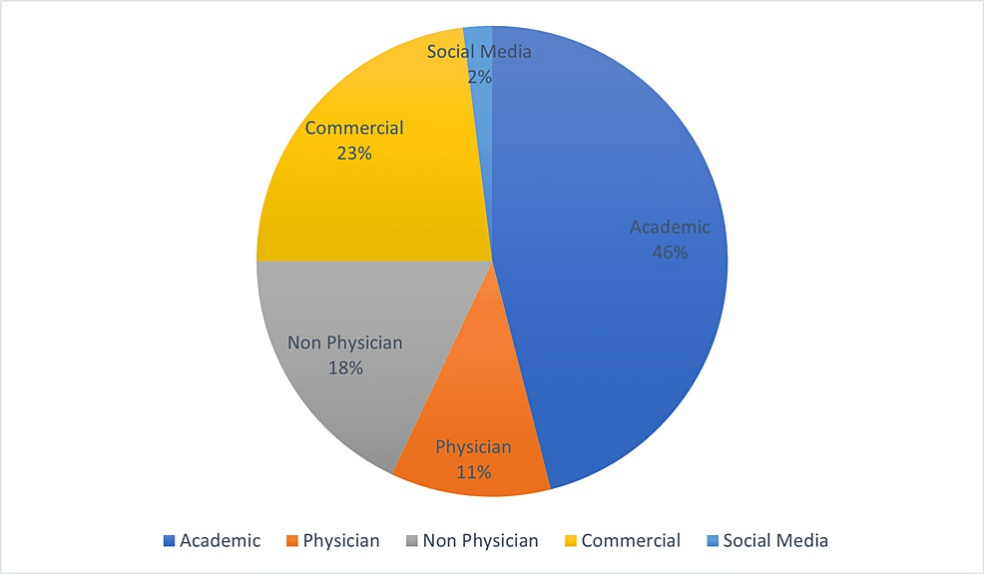
Authorship classification according to affiliation

Authorship was then categorized into academic, physician, non-physician, commercial, and social media groups (Figure 2). Quality assessment was done by two independent authors using the validated tools DISCERN, the JAMA benchmark criteria, and HON Code certification within two weeks of the original search [14-16]. DISCERN is a tool, which has been designed to help users of consumer health information judge the quality of written information about treatment choices [14]. It's a brief questionnaire that evaluates the reliability of publication, the quality of information about treatment choices, and the overall quality of the publication. The JAMA benchmark criteria assess the following four core standards: website authorship (authors, contributors, affiliations, and credentials), attribution (references and sources used for the content, copyright information), disclosures (sponsorship, advertising, commercial funding, potential conflicts of interests), and currency (dates of posted and updated information) [15]. A list of all websites can be found in Appendix 1.

The readability score was calculated using online software tools, www.readable.com and www.webfx.com [17-18]. The three scores calculated were FRES, FKG, and GFI. These scores indicate the level of education needed to comprehend the text with ease. Four websites could not be analyzed using webfx.com for which www.seoreviewtools.com was used. Where URLs couldn't be analyzed, the text from the website was used to calculate readability scores.

## Results

Quality analysis

**Figure 3:**
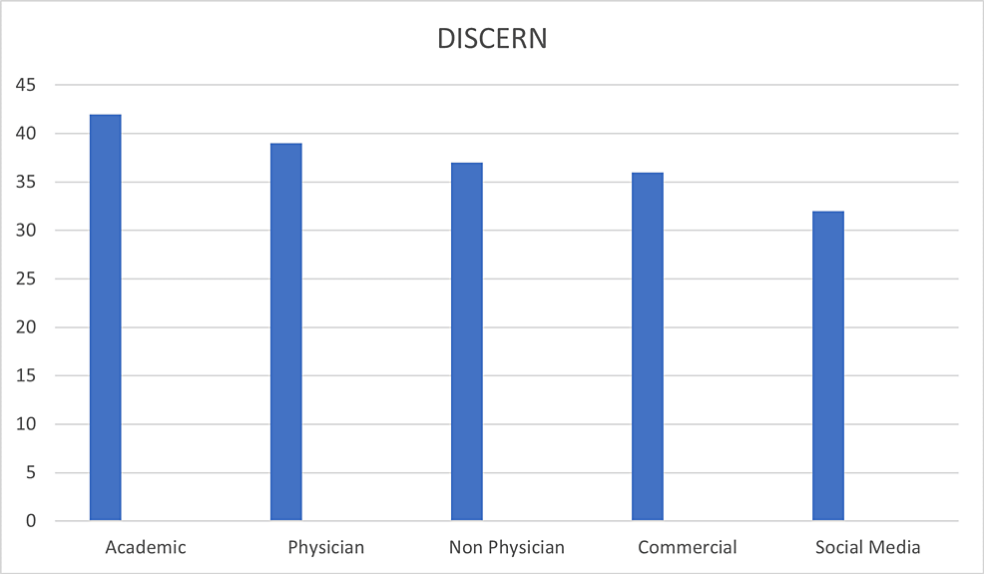
DISCERN readability data Quality analysis showing means scores for individual groups

The mean DISCERN score was 39.55 (26-66). We also noted that academic websites had a higher DISCERN score as compared to others. The Health on the Net Foundation (HON) code-certified sites also scored high on the DISCERN tool (Figure 3). There were only 16 HON code-certified websites out of a total of 83 (19%).

**Figure 4:**
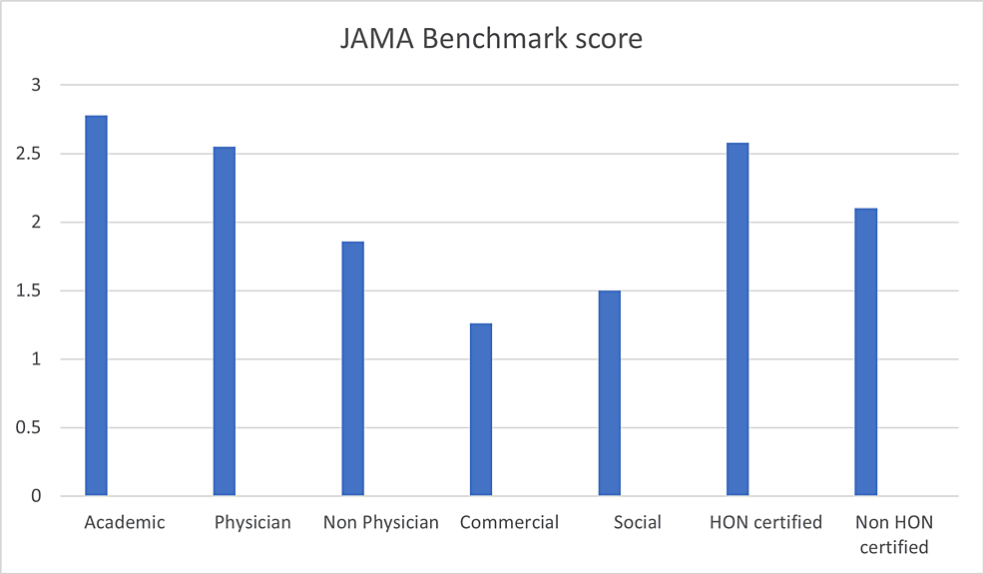
JAMA benchmark score JAMA mean scores according to affiliation and HON certification JAMA: Journal of the American Medical Association

The mean JAMA score was 2.21 (1-4) (Table 1). Fourteen websites fulfilled all four JAMA benchmark criteria while a substantial number of 31 scored only one. Sixty-eight percent (68%; n 11) of HON code-certified websites also scored 3 or 4 on the JAMA scale (Figure 4).

Readability analysis

**Figure 5:**
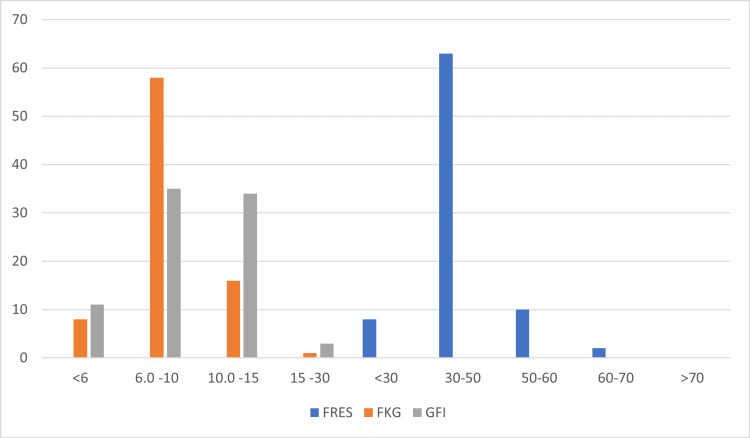
Readability data FKG: Flesch-Kincaid Grade; FRES: Flesch Reading Ease Score; GFI: Gunning Fog Index

The mean Flesch readability ease score (FRES) noted was 49.26 (14.1-110.3), mean Flesch-Kincaid grade level (8.38; - 0.2 - 18.5), and mean Gunning Fog index (9.51, 2.1 - 18.5) (Table 1). Only two websites had an FRE score of > 60, which corresponds to eighth-grade level. The majority of the 63/83 participants were at the college level (FRES 30-60). Eight out of 83 scored less than 30, corresponding to increasing difficulty in reading and understanding (Figure 5).

**Table 1:** Overall results for each assessment tool DISCERN: DISCERN Instrument; FKGL: Flesch-Kincaid Grade Level; FRES: Flesch Reading Ease Score; GFI: Gunning Fog Index; HON: Health on the Net Foundation; JAMA: The Journal of the American Medical Association benchmark criteria; n: number; r: range

Category	No of sites	JAMA Mean (range)	DISCERN Mean (range)	HON code-certified	FRES Mean (range)	FKG Mean (range)	GFI Mean (range)
Academic	38	2.7 (1-4)	42.2 (26–66)	6	47.5 (14.1–108.8)	8.6 (1.1–18.5)	9.7 (2.4–18.5)
Physician	9	2.5 (1-4)	39.5 (28-54)	6	57.3 (34.8-110.3)	7.1 (-0.2-10.8)	8.4 (3.4-11.8)
Non Physician	15	1.8 (1-3)	37.1 (26-52)	2	50.9 (37.8-91.8)	7.9 (1-11.9)	9.1 (2.1-15.7)
Commercial	19	1.2 (1-3)	36.7 (29-53)	2	46.7 (21.3-62.3)	8.6 (6.4-11.3)	9.7 (6.8-11.9)
Social Media	2	1.5 (1-2)	32.5 (29-36)	-	55.7 (55-56.5)	9.4 (9.2-9.6)	10.5 (10.1-11)
Total	n = 83	Mean 2.2	Mean 39.5	n = 16	Mean 49.2	Mean 8.3	Mean 9.5

Thirty-three (33) websites had an FKG value of < 8. Out of these, seven scored less than six, mirroring the sixth-grade reading level. A total of 17/83 had a score of > 10, suggesting a tenth-grade level (Figure 5). The mean Gunning Fog Index score was 9.13, which is more than the recommended level of 7. There was no major readability score difference between HON code-certified and non-certified websites. Table 1 shows the overall results for each assessment tool.

## Discussion

Over the past few years, life expectancy has increased by several years [19]. The key challenge is not only to extend the life span but also to ensure those extra years are healthy and disability-free for as long as possible.

To date, there have been no studies assessing the information available to the general public on the Internet regarding osteoporotic vertebral compression fractures. The lack of health literacy is associated with increased health disparities, poor health outcomes, and increased hospital admissions, among other health care safety issues, including medical errors and medication errors [20]. In America, health literacy has been identified as one of the 20 necessary actions to be taken to enhance health care quality [21-22].

Patients had historically been limited to a few medical information sources, most of which were written for health professionals. As a result of recent trends in society, such as the predominant internet and the need for patients to be informed "consumers of health care services," a wide variety of health information is now available [23]. A reliable source of health information can be of great value to patients who make routine minor and major health decisions as the informed patient is more equipped to participate in a conversation about treatment alternatives, and recent research has shown that when patients participate in decision-making, they have better subjective results.

In the present study, the material available on the Internet is very varied and of low to moderate quality on health concerns. There were discrepancies in the authorship categories on the DISCERN score and JAMA benchmark criteria, with physician-compiled and academic websites offering higher-quality content followed by other categories. Despite evidence that health education material is best provided at the fifth or sixth-grade level, our data repeatedly showed that information on osteoporotic vertebral compression fractures on the Internet is written at roughly the tenth to twelfth-grade level. Our study's mean FRES, FKG, and GFI scores were 49.26, 8.38, and 9.51 respectively, which are much higher than the recommended sixth-grade reading level as advocated by the American Medical Association (AMA). Only 16% of websites had a FRES score of > 60 at par with the eighth-grade level while 8.4% had an FKG score below 6. One of the reasons for having high readability scores is the fact that 56% of websites are either academic or physician-compiled as stated previously.

Our study's limitations are that we cannot tailor recommendations to specific segments of the patient population. Recent research has shown that there is a lot of variation in internet access, regardless of age, ethnicity, income, or level of education [24]. Future research should focus on how access to the Internet affects search habits and search term complexity within different segments of the population. Another potential limitation of the study is that the reliability of the display of the HON code [25] certification itself has been questioned, and our study was unable to parse out fraudulent uses of the HON code logo. Finally, although we searched three keywords across five search engines, the results were confined to the first 50 websites per search engine.

## Conclusions

The main highlight of this study has been the low quality and comprehensibility of online information on osteoporotic vertebral fractures. There is a paucity of easily readable and accessible websites for an average person, which can potentially affect patient outcomes. The need of the hour is to have reliable, readable, and quality online resources available regarding common health problems to maximize patient satisfaction and outcomes.

## Appendices

**Table 2:** Supplementary data

1	https://www.physiopedia.com/Osteoporotic_Vertebral_Fractures#:~:text=Osteoporotic%20vertebral%20fractures%20are%20fractures,and%20more%20likely%20to%20fracture
2	https://www.webmd.com/osteoporosis/guide/osteoporosis-and-spine-fracture
3	https://orthoinfo.aaos.org/en/diseases--conditions/osteoporosis-and-spinal-fractures/
4	https://www.orthobullets.com/spine/2021/osteoporotic-vertebral-compression-fracture
5	https://bestpractice.bmj.com/topics/en-us/819
6	https://www.aans.org/en/Patients/Neurosurgical-Conditions-and-Treatments/Vertebral-Compression-Fractures
7	https://theros.org.uk/information-and-support/osteoporosis/spinal-fractures/
8	https://www.spine-health.com/conditions/osteoporosis/when-back-pain-a-spine-compression-fracture
9	https://www.health.harvard.edu/newsletter_article/treating_osteoporotic_fractures_of_the_spine
10	https://emedicine.medscape.com/article/325872-overview
11	https://www.spineuniverse.com/conditions/osteoporosis/osteoporosis-compression-fractures
12	https://www.aafp.org/afp/2016/0701/p44.html
13	https://www.uptodate.com/contents/osteoporotic-thoracolumbar-vertebral-compression-fractures-clinical-manifestations-and-treatment
14	https://www.ajronline.org/doi/10.2214/ajr.183.4.1830949
15	https://qims.amegroups.com/article/view/29619/25730
16	https://www.dovepress.com/investigation-of-preoperative-traction-followed-by-percutaneous-kyphop-peer-reviewed-fulltext-article-IJGM
17	https://radiopaedia.org/articles/spinal-compression-fracture
18	https://my.clevelandclinic.org/health/diseases/17498-spinal-fractures
19	https://www.sciencedirect.com/science/article/pii/S2214031X18300986fractures
20	https://www.nuffieldhealth.com/conditions/osteoporotic-vertebral-
21	https://www.osteoporosis.foundation/health-professionals/fragility-fractures/treatment
22	https://www.msdmanuals.com/professional/injuries-poisoning/fractures/vertebral-compression-fractures
23	https://www.medi.de/en/diagnosis-treatment/osteoporosis/vertebral-fracture/
24	https://www.aomrc.org.uk/ebi/clinicians/vertebral-augmentation-vertebroplasty-or-kyphoplasty-for-painful-osteoporotic-vertebral-fractures/
25	https://www.umms.org/ummc/health-services/orthopedics/services/spine/patient-guides/thoracic-compression-fractures
26	https://stanfordhealthcare.org/medical-conditions/back-neck-and-spine/osteoporotic-fractures.html
27	https://www.cedars-sinai.org/health-library/diseases-and-conditions/c/compression-fracture.html
28	https://www.spinal-foundation.org/conditions/osteoporosis-fracture
29	https://www.ncbi.nlm.nih.gov/pmc/articles/PMC6583415/
30	https://gpnotebook.com/simplepage.cfm?ID=-1523253245
31	https://theros.org.uk/media/3daohfrq/ros-vertebral-fracture-guidelines-november-2017.pdf
32	https://revistadeosteoporosisymetabolismomineral.com/2017/07/11/epidemiology-of-osteoporotic-fractures-vertebral-and-non-vertebral-fractures/
33	https://findanyanswer.com/what-is-considered-a-major-osteoporotic-fracture
34	https://probolezny.ru/osteoporoz/
35	https://en.wikipedia.org/wiki/Osteoporosis
36	https://www.hindawi.com/journals/prm/2020/3947368/
37	https://www.physio-pedia.com/Thoracic_Spine_Fracture
38	https://radiologykey.com/vertebral-fractures-and-osteoporosis/
39	https://diseases.medelement.com/disease/%D0%BE%D1%81%D1%82%D0%B5%D0%BE%D0%BF%D0%BE%D1%80%D0%BE%D0%B7-%D0%BA%D0%BF-%D1%80%D1%84-2021/16662
40	https://www.epainassist.com/back-pain/vertebral-fracture
41	https://www.versusarthritis.org/about-arthritis/conditions/osteoporosis/
42	https://www.medicinenet.com/osteoporosis/article.html
43	https://www.nhs.uk/conditions/osteoporosis/
44	https://www.guidelines.co.uk/musculoskeletal-and-joints-/osteoporotic-fracture-guideline/455546.article
45	https://jensenstore.com/osteoporotic-vertebral-fractures/
46	https://asbmr.onlinelibrary.wiley.com/doi/full/10.1002/jbmr.3669
47	https://academic.oup.com/bmb/article/102/1/171/310445?login=false
48	https://www.southtees.nhs.uk/content/uploads/Vexim-Patient-Brochure.pdf
49	https://www.cheltenhamspineclinic.co.uk/osteoporotic-vertebral-compression-fractures-vcf/
50	https://www.mayoclinic.org/diseases-conditions/osteoporosis/symptoms-causes/syc-20351968
51	https://www.healthline.com/health/compression-fractures-of-the-back
52	https://www.joint-surgeon.com/back-and-spine-specialist/kyphoplasty-minimal-invasive-back-and-spine-procedure
53	https://www.beaumont.org/treatments/vertebral-compression-fracture-treatment
54	https://www.uclahealth.org/neurosurgery/osteoporotic-vertebral-fractures
55	https://www.uscspine.com/conditions-treated/back-disorders/osteoporosis-compression-fractures/
56	https://www.nbt.nhs.uk/sites/default/files/attachments/Spinal%20fractures_NBT03193.pdf
57	https://www.orthovirginia.com/blog/osteoporosis-and-spinal-compression-fracture
58	https://www.mumbaispineclinic.com/spinal-osteoporosis/
59	https://isass.org/for-patients/spine-conditions/osteoporosis-and-vertebral-fractures/
60	https://drgavinclunie.co.uk/blog/all-about-vertebral-fractures
61	https://www.orthomaryland.net/specialties/Spine-Care/spinal-compression-fractures
62	https://www.hopkinsmedicine.org/health/conditions-and-diseases/compression-fractures
63	https://www.neurotexas.net/conditions/spinal-disorders/osteoporosisspinal-fractures/
64	https://www.bonehealthandosteoporosis.org/patients/treatment/exercisesafe-movement/osteoporosis-and-your-spine/
65	https://www.topdoctors.co.uk/medical-dictionary/osteoporosis-of-the-spine
66	https://curejoyinc.com/content/osteoporosis-and-spinal-fractures/
67	https://www.csp.org.uk/conditions/falls-fractures
68	https://utswmed.org/conditions-treatments/fragility-fracture/
69	https://lesann.tripod.com/fragility%20fractures.htm
70	https://www.med.cam.ac.uk/poole/having-a-fracture/
71	https://www.bsir.org/patients/vertebral-compression-fractures/
72	https://oahct.com/direct-orthopedic-care/trauma-and-fractures-orthopedic-specialist/fragility-fractures/
73	https://www.boneandjointburden.org/fourth-edition/iva2/fragility-fractures
74	https://patient.info/doctor/fragility-fractures
75	https://ukspinecentre.co.uk/osteoporotic-vertebral-compression-fractures/
76	https://vicorthospine.com.au/conditions/osteoporotic-compression-fractures/
77	https://medicalxpress.com/news/2010-10-devastating-impact-spinal-osteoporotic-fractures.html
78	https://www.betterbones.com/fractures-and-healing/fracture-types/
79	https://age2b.com/spinal-fractures/
80	https://sci.washington.edu/info/forums/reports/osteoporosis.asp
81	https://www.leedsspineclinic.co.uk/spine-fractures.html
82	https://www.fracturedtruths.com/osteoporosis-fracture-facts
83	https://elvizgasimov.com/en/pathological-fractures-of-the-spinal-column-symptoms
